# Multifaceted Effects of Extracellular Adenosine Triphosphate and Adenosine in the Tumor–Host Interaction and Therapeutic Perspectives

**DOI:** 10.3389/fimmu.2017.01526

**Published:** 2017-11-14

**Authors:** Paola de Andrade Mello, Robson Coutinho-Silva, Luiz Eduardo Baggio Savio

**Affiliations:** ^1^Division of Gastroenterology, Department of Medicine, Beth Israel Deaconess Medical Center, Harvard Medical School, Boston, MA, United States; ^2^Instituto de Biofísica Carlos Chagas Filho, Universidade Federal do Rio de Janeiro, Rio de Janeiro, Brazil

**Keywords:** purinergic signaling, P2X7 receptor, CD39, CD73, tumor microenvironment, immunotherapy

## Abstract

Cancer is still one of the world’s most pressing health-care challenges, leading to a high number of deaths worldwide. Immunotherapy is a new developing therapy that boosts patient’s immune system to fight cancer by modifying tumor–immune cells interaction in the tumor microenvironment (TME). Extracellular adenosine triphosphate (eATP) and adenosine (Ado) are signaling molecules released in the TME that act as modulators of both immune and tumor cell responses. Extracellular adenosine triphosphate and Ado activate purinergic type 2 (P2) and type 1 (P1) receptors, respectively, triggering the so-called purinergic signaling. The concentration of eATP and Ado within the TME is tightly controlled by several cell-surface ectonucleotidases, such as CD39 and CD73, the major ecto-enzymes expressed in cancer cells, immune cells, stromal cells, and vasculature, being CD73 also expressed on tumor-associated fibroblasts. Once accumulated in the TME, eATP boosts antitumor immune response, while Ado attenuates or suppresses immunity against the tumor. In addition, both molecules can mediate growth stimulation or inhibition of the tumor, depending on the specific receptor activated. Therefore, purinergic signaling is able to modulate both tumor and immune cells behavior and, consequently, the tumor–host interaction and disease progression. In this review, we discuss the role of purinergic signaling in the host–tumor interaction detailing the multifaceted effects of eATP and Ado in the inflammatory TME. Moreover, we present recent findings into the application of purinergic-targeting therapy as a potential novel option to boost antitumor immune responses in cancer.

## Introduction

Cancer is still one of the world’s most pressing health-care challenges, leading to death in an estimated number of 600,920 patients per year in the United States (1). However, recent advances in cancer immunotherapy have transformed the treatment of several patients, extending and improving their lives (2, 3). Immunotherapy is a new developing therapy that boosts patient’s immune system to fight cancer, by modifying tumor–immune cells interaction in the tumor microenvironment (TME) (4). According to the cancer immunoediting concept, the interaction between cancer and immune cells occurs in three essential phases: elimination, equilibrium, and escape—from cancer immune surveillance to immune escape (5–7). In the elimination and equilibrium phase innate and adaptive immune system—mainly NK and T cells—mount an effective immune response against the highly immunogenic tumors, and allow the less immunogenic ones escape (8–16). This immunologic pressure selects and favors tumor variants resistant to the immune system to proliferate (immunoevasion) (9, 17). During this process, both cancer and inflammatory cells release several soluble factors such as cytokines, chemokines, growth factors, matrix-degrading enzymes, and nucleotides that facilitate tumor immune escape and allow tumor growth, angiogenesis, invasion, and metastasis (18–22). Therefore, targeting multiple molecules that avoid immunoevasion and boost antitumor immune responses are the leading paths to successfully treat a whole range of tumor types (3).

Among the nucleotides released in the TME, extracellular adenosine triphosphate (eATP) and adenosine (Ado) are potent modulators of both immune and tumor cell response (23, 24). eATP and Ado exert their effects acting through P2 and P1 purinergic receptors, respectively, triggering the so-called purinergic signaling (25, 26). Purinergic signaling has long been involved with inflammation and cancer having a pivotal role in modulating cell migration, proliferation, and cell death (27, 28). P2 and P1 receptors are expressed by nearly all cell types (immune and non-immune cells) and differently trigger cell signaling according to their subtypes (29–31). The P2 receptor is subdivided into two separate subfamilies, P2X (P2X1–7) ionotropic ion channels receptors and P2Y (P2Y1, P2Y2, P2Y4, P2Y6, and P2Y11–P2Y14) G-protein-coupled receptors (25, 26), whereas the P1 receptor family (A_1_, A_2A_, A_2B_, and A_3_) only comprised by G-protein-coupled receptors subtype (32). These different purinergic receptors express distinct agonist affinity and specificity, therefore influencing both tumor and immune cells behavior according to the levels of eATP/Ado in TME (33–35).

Levels of eATP and Ado are tightly controlled by several ectonucleotidases. Among them, CD39 and CD73 are the most important ecto-enzymes expressed in cancer cells, regulatory immune cells and vasculature responsible for modulating purinergic signaling within the TME (36, 37). CD39 is a member of the ectonucleoside triphosphate diphosphohydrolase (E-NTPDase) family that comprised of eight members (E-NTPDase1–8), each one with a distinct cellular location and catalytic properties (36, 37). E-NTPDase1 (CD39), E-NTPDase2, E-NTPDase3, and E-NTPDase8 are plasma membrane-bound enzymes that degrade with different affinities adenosine triphosphate (ATP) and ADP to AMP (24, 36, 37). AMP is in turn converted to Ado by CD73, which is an ecto-5′-nucleotidase cell-surface enzyme (37). This sequential activity of CD39/CD73 is the main pathway for the eATP scavenging and generation of Ado in the tumor interstitium (24, 36).

Once accumulated in the TME, eATP and Ado act as signaling molecules triggering different and opposite effects on both host and tumor cells. While eATP boosts antitumor immune response and Ado attenuates or suppresses immunity on the host side (38–45), both molecules can mediate growth stimulation or inhibition on the tumor cells, depending on the specific receptor activated (46–52). Regardless, the final effect on tumor growth—either beneficial or detrimental—will depend on the eATP/Ado levels, the panel of P2 and P1 receptors subtypes and CD39/CD73 expression by immune, tumor, and stromal cells in the TME (22).

Therefore, despite its complexity and dual behavior, modulation of purinergic signaling by targeting eATP/Ado pathways appears to be a promising strategy to modify cancer and immune cells cross talk in the TME (24, 36, 53). In this review, we will discuss the role of purinergic signaling into the host–tumor interaction detailing the multifaceted effects of eATP and Ado in the inflammatory TME. Furthermore, we will highlight the application of combining purinergic-targeting therapies with other anticancer treatments as a potential new strategy to overcome immune escape, potentiate antitumor immune response, and, consequently, restrain tumor growth.

## eATP in the TME

Measurement of eATP levels in different biological context reveals that healthy tissues present very low levels (10–100 nM) of this nucleotide in the pericellular space, while in sites of tissue damage, inflammation, hypoxia, ischemia, TME or metastases it can reach high levels (hundreds of micromoles per liter) (24, 54–56). ATP is abundantly released in the extracellular space due to cell death, cell stress, and activation of pannexin/connexin channels on immune and endothelial cells (54, 57, 58). In these settings, increased levels of eATP are sensed as a “danger signal” by the innate immune cells resulting in their recruitment to the damaged-tissue site (42, 57, 59–61). Particularly in the TME, eATP acting through P2 receptors boosts the antitumor immunity at the same time that stimulates endothelial and tumor cells (27, 36, 42, 48, 60).

### eATP Effect on the Host Side

Activation of P2 receptors by eATP shapes various innate and adaptive immune responses (30). The P2X and P2Y receptors expression (either constitutive or upregulated in pathological conditions) varies according to the cell type and therefore dictates immune cell function, such as metabolism, adhesion, activation, migration, maturation, release of inflammatory mediators, cytotoxicity, and cell death, as extensively reviewed in Ref. (30, 36, 62). In the innate immunity, activation of P2Y_2_ and P2X7 receptors leads to stimulation of myeloid cells and promotes chemotaxis of macrophages and neutrophils (38, 63–65). At the same time, engagement of P2Y_2_ and P2X7 receptors induces dendritic cells (DCs) activation and chemotaxis (66). Indeed, stimulation of P2Y_11_ receptor inhibits IL-12 and boosts IL-10 release by DCs (67) whereas it activates granulocytes (68). In the adaptive immunity, engagement of various P2X receptors, such as P2X1, P2X4, P2X5, and P2X7, results in T-cell activation (39, 69–71). Among them, P2X7 has been linked to stimulation of CD4^+^ and CD8^+^ effector T cells (40, 69, 72) as well as NKT cells (73), induction of Treg apoptosis (41, 74, 75), and inhibition of Tr1 cell differentiation (76). In addition, ATP acting *via* the P2X7 receptor is crucial to the generation of inflammatory Th17 lymphocytes by contributing for the generation of a microenvironment with high levels of IL-1β, IL-6, and IL-17 (77, 78).

In the context of TME, recent studies have highlighted the importance of eATP acting through the P2X7 receptor in the chemotherapy-elicited anticancer immune response, also known as immunogenic cell death (ICD) (42, 60). Accordingly, ATP derived from dying tumor cells stimulates P2X7 receptors in DCs, thus activating the NLRP3/ASC/caspase-1 inflammasome and driving the secretion of interleukin-1β (IL-1β). IL-1β is then required for the adequate polarization of IFNγ-producing CD8^+^ T cells, which is critical for the efficacy of chemotherapy (42, 60).

Despite its role in ICD, eATP-P2X7 signaling has also been related to the control of tumor growth. Recent studies have shown that host P2X7 expression limits tumor growth and metastasis spread by supporting an antitumor immune response (47, 79). Host P2X7 seems to boosts cytokine release, chemotaxis, and tumor infiltration by inflammatory cells. Accordingly, P2X7 host genetic deletion in mouse (P2X7-KO) impaired immune response against melanoma (B16) and colon carcinoma cells (CT26), leading to accelerate tumor growth in comparison to P2X7-WT hosts. Moreover, transplantation of P2X7-WT bone marrow to P2X7-KO mice reduced tumor growth at a rate similar to the P2X7-WT group (47).

Even though eATP acting through P2X7 receptor seems to be an important signaling to stimulate immune cell response against the tumor, a critical role for the ATP/P2X7 receptor axis in modulating myeloid-derived suppressor cells (MDSCs) functions in the TME has also been described (23). Accordingly, P2X7 receptor activation stimulates the release of reactive oxygen species, arginase-1, and transforming growth factor-β 1 (TGF-β1) from monocyte MDSCs present in the TME, contributing to MDSC immunosuppressive effect. Therefore, considering these contradictory effects the use of both antagonist/agonist of the P2X7 receptor has been investigated as a promising novel strategy for anticancer therapy and will be discussed with more details below.

### eATP Effect on the Tumor Side

Practically all types of cancer cells express P2X and P2Y receptors that efficiently sense changes in ATP concentration in the TME and modulate different cellular functions such as proliferation, differentiation, and apoptosis (24, 28). Cancer cells may be more sensitive to the cytotoxic or to the trophic effect of e ATP according to the expression of their P2 receptor subtypes as well reviewed in Ref. (28).

Among the P2Y receptors, stimulation of P2Y_2_ and P2Y_11_ receptors leads to cell proliferation and migration of human hepatocellular carcinoma (HCC) cells (49, 80). P2Y_2_ receptor activation is also highly involved with tumor invasiveness and metastatic diffusion in prostate and breast cancer (81–87). On the other hand, eATP-P2Y_2_ receptor signaling inhibited nasopharyngeal carcinoma and human colon carcinoma growth (50, 88). P2Y_1_ receptor activation induces apoptosis and inhibits human intestinal epithelial carcinoma, prostate cancer, and melanoma cell proliferation (89–91).

In the P2X receptors family, a role for P2X3, P2X5, and P2X7 in carcinogenesis has already been depicted, with a major focus on the P2X7 receptor. P2X3 receptor overexpression seems to be crucial for HCC cell survival and basal proliferation as well as proliferation in response to changes in ATP concentrations in the TME (92). Moreover, high P2X3 receptor expression is associated with poor prognosis in patients with HCC. P2X5 overexpression was also demonstrated in human basal cell and squamous carcinomas, but differently, it was expressed exclusively on cells undergoing proliferation and differentiation, suggesting a different role in tumor growth (93).

**Figure 1:**
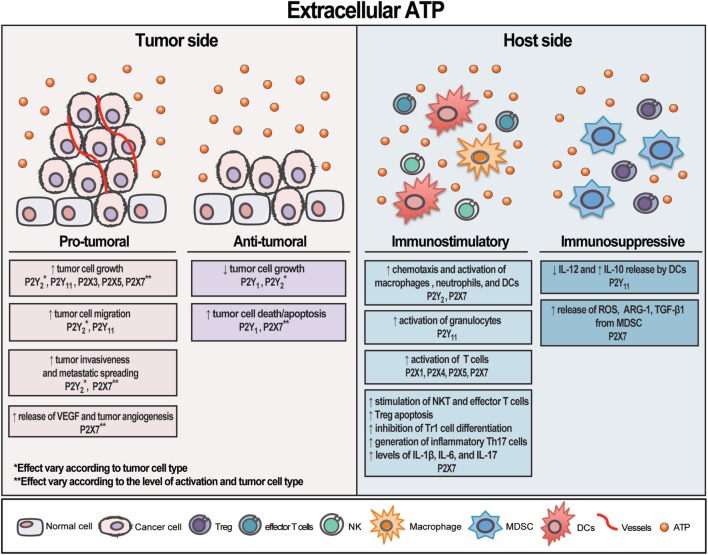
Schematic illustration showing extracellular adenosine triphosphate (eATP) contrasting effects on tumor and host side. eATP can trigger different and opposite effects on both tumor and host cells depending on the cell type and receptor activated. The final result—either stimulating or restraining tumor growth—will depend on the eATP levels, the panel of P2 receptor subtypes and CD39/CD73 expression by tumor and immune cells present in the tumor microenvironment. Overall, eATP is a potent pro-inflammatory mediator, mostly boosting immune cells response.

P2X7 is far the most P2X receptor subtype studied in cancer. Unlike the other P2 receptors, P2X7 is unique for its capacity to form a nonselective pore on the plasma membrane upon stimulation with high levels of eATP, leading to cell death (94, 95). Its role in carcinogenesis remains a controversy, but now it is known that P2X7 receptor triggers cell death or growth according to its level of activation and cell type stimulated (94, 96–98). As mentioned earlier, P2X7 receptor overstimulation with a high level of exogenous eATP triggers tumor cell death, while its tonic stimulation with endogenous eATP often induces cancer cell survival and proliferation (28, 99, 100). Whereas the former leads to a marked mitochondrial catastrophe, the latter stabilizes the mitochondrial network, increases mitochondrial potential, oxidative phosphorylation, and aerobic glycolysis, culminating in a large increase in the overall intracellular ATP content and gain in proliferative advantage by P2X7-expressing cells (99). P2X7 receptor activation also triggers NFATc1, Erk, PI3K/Akt, and HIF-1α intracellular pathways (101–103), being the PI3K/Akt pathway linked to the P2X7-dependent tumor cell growth, invasiveness, metastatic spreading, and angiogenesis (101, 104). Also supporting a role for P2X7 receptor in tumor growth is the fact that many types of cancer such as leukemia (98, 105, 106), melanoma (107), neuroblastoma (108), pancreatic adenocarcinoma (109), esophageal carcinoma (110), breast (111), prostate (112), thyroid (113), and head and neck cancer (114) showed an increased expression of P2X7 receptor. Moreover, *in vivo* experiments demonstrated that blocking P2X7 receptor activation by either silencing or a pharmacological manipulation decreased tumor progression and inhibited metastatic diffusion (100, 115). Therefore, it seems reasonable to say that P2X7 receptor is an important target in cancer therapy not only for its role in the immune system but also for its impact on tumor growth. An overview of eATP effect on tumor and host side is illustrated in Figure 1.

## eADENOSINE in the TME

High levels of extracellular adenosine (eAdo) were also demonstrated in the TME. While Ado levels in healthy tissue are around the nanomolar range, it can reach the micromolar range in the tumor core (36, 51, 116, 117). In the later context, many factors can contribute to Ado production, but hypoxia seems to be the main driver for the eAdo accumulation (118). In this setting, eAdo is mainly generated at the expenses of the eATP metabolism *via* the sequential enzymatic activity of CD39 and CD73 (119–122). CD39 catalyzes the first enzymatic reaction by breaking down ATP and ADP into AMP, whereas CD73 hydrolyzes AMP into Ado. CD73 irreversibly converts AMP to Ado being considered the rate-limiting enzyme for Ado formation (37, 122).

Many cells have the capacity to generate eAdo in the TME, such as tumor cells (43, 120, 123–126), Tregs (120, 127, 128), Th17 (129, 130), MDSCs (44, 131, 132), endothelial cells (127, 133, 134), cancer-associated fibroblast (135, 136), and mesenchymal stromal/stem cells (MSCs) (45, 137). Exosomes derived from CD39^+^CD73^+^ tumor cells (138), Tregs (139), or MSCs (45) can also contribute to eAdo production. Once in the pericellular space, Ado can exert a local signaling effect through the activation of the P1 purinergic receptors, be metabolized to inosine or recaptured by the cell *via* nucleoside transporters (140).

Likewise eATP, eAdo acts as an endogenous immunomodulatory molecule, but unlike the former, it mostly mediates immunosuppressive effects (30). Particularly in the tumor interstitium, eAdo acting through P1 receptors downregulates cell-mediated immunity at the same time that stimulates tumor cells and promotes angiogenesis (45, 133, 136, 137).

### eAdo Effect on the Host Side

Extracellular adenosine exerts immunosuppressive activities in various immune subsets, interfering with antitumor immune responses (36). Innate and adaptive immune cells react to Ado stimulation according to the expression/density of the four P1 receptor subtypes, namely A_1_, A_2A_, A_2B_, and A_3_ (30, 32). These receptors sense different levels of Ado and are classified as high-affinity (A_1_, A_2A_, and A_3_) and low-affinity receptors (A_2B_) (32). A_1_ and A_3_ are Gi-coupled receptors that inhibit adenylate cyclase and cyclic AMP production, while A_2A_ and A_2B_ are Gs-coupled receptors that stimulate cAMP synthesis and downstream signaling pathways (32, 141).

Activation of A_2A_ and A_2B_ receptors protect tissues against excessive immune reaction and therefore play a major role in Ado immunosuppressive effects (142–146). Stimulation of A_2A_ receptor is related to the inhibition of DC activation (147), Th1/Th2 cytokine production (148, 149), T cells proliferation and activation (148, 149), and NK cells activation, maturation, and cytotoxicity (125, 150), as well as enhancement of the suppressive function of Tregs, Tr1 cells, and macrophages (151–153). In addition, A_2A_ receptor activation prevents the LPS-induced increase in ectonucleotidase activities during inflammation (154, 155).

Activation of the A_2B_ receptor has a major effect on Tregs and MDSCs, stimulating Treg proliferation or differentiation from naïve T cells, production of IL-10 (156) and enhancing the suppressive function of MDSCs (44). A_2B_ signaling is also linked to vascular endothelial growth factor (VEGF) secretion and tumor angiogenesis (44, 157). Engagement of A_2A_ and A_2B_ receptors inhibits neutrophils activation (158) and immune cells adhesion to endothelial cells (127). On the other hand, activation of A_1_ and A_3_ receptors promotes neutrophils chemotaxis and stimulates pro-inflammatory activities (158).

In general, Ado accumulation in the TME and its immunosuppressive effect *via* A_2A_ and A_2B_ receptors is a critical regulatory mechanism implemented by the tumors to evade the immune-mediated cancer cells destruction, allowing tumor growth and impairing cancer immunosurveillance (159). In this way, new strategies targeting Ado production and signaling have emerged as a promising approach in cancer immunotherapy and will be discussed in more details below.

### eAdo Effect on the Tumor Side

Differently from its effect on the host side, where Ado is well known for its strong immunosuppressive activities, on the tumor side Ado can either stimulate or inhibit tumor growth, depending on the cell type and receptor expressed by the tumor bulk (160). Likewise, pro- and antitumoral effects coming from A_1_, A_2A_, A_2B_, and A_3_ activation have been described (160). A_1_ receptor activation is related to stimulation of MDA-MB-468 breast carcinoma cells proliferation (161) and melanoma cells chemotaxis (162). On the other hand, it may inhibit LoVo colon (163), TM4 Sertoli-like (164), MOLT-4 leukemia, T47D, HS578T, and MCF-7 breast, and glioblastoma cancer cells proliferation (160). Ado-A_1_ signaling has also been reported to protect endometrial carcinoma invasion and metastasis, by promoting cortical actin polymerization, increasing cell–cell adhesion thus preserving epithelial integrity (165). In the same manner, activation of A_2A_ and A_2B_ receptors leads to controversial scenarios depending on the cell type studied. A_2A_ stimulation results in increased MCF-7 breast cancer proliferation (166), whereas it promotes A375 melanoma cell death (167). Activation of A_2B_ receptor inhibits ER-positive MDA-MB-231 breast cancer cell proliferation, while it boosts oral squamous cell carcinoma progression (168, 169). Stimulation of A_2B_ receptor also leads to reduced cell–cell contact and increased cell scattering in breast, lung, and pancreatic cancer cell lines, suggesting a role for this receptor in tumor invasion and metastatic spreading (170). These conflicting results might reflect differences in the experimental settings where distinct tumor cell lines were exposed to diverse agonist/antagonist drugs with different specificity and selectivity. Moreover, the use of specific agonist might not reflect the real effect triggered by Ado in the context of the tumor bulk given the complexity and heterogeneity of cells, Ado receptors, and downstream signaling that interact to produce the final cellular response.

A_3_ is by far the most studied Ado receptor in cancer and conflicting results have also been reported for this receptor. A_3_ receptor is expressed by many tumor cell lines, such as HL60 and K562 human leukemia (171, 172), Jurkat lymphoma (173), U937 monocytic–macrophagic human cell lines (174, 175), Nb2 rat lymphoma (176), A375 human melanoma (177), PGT-betamouse pineal gland tumor cells (178), human glioblastoma (179, 180), and human prostatic cancer cells (181). Moreover, A_3_ overexpression (either protein or mRNA levels) has been reported in human melanoma, colon, breast, small-cell lung, thyroid, pancreatic, and HCC vs adjacent normal tissue, supporting the notion that A_3_ receptor levels may reflect the status of tumor progression (182–184). In accordance with this statement, A_3_ activation increases HT29, DLD-1 and Caco-2 colon cancer cell proliferation (160). However, A_3_ stimulation also results in antitumoral effects, inhibiting proliferation of Nb2-11C and YAC-1 lymphoma, K562 and HL60 leukemia, B16-F10 and A375 melanoma, LN-Cap and PC3 prostate carcinoma, MIA-PaCa pancreatic carcinoma, breast and Lewis lung carcinoma cells (176, 185–189). Contrasting responses were also reported for A_3_ stimulation on metastatic spreading, leading to either increased (HT29 colon carcinoma) or decreased (prostatic cancer) cell migration (179, 181). Despite these dual effects, the A_3_ receptor has been pointed as a potential target for tumor growth inhibition (182, 190). A phase I/II clinical trial using an A_3_ agonist for the treatment of advanced unresectable HCC has been performed and despite preliminary data, favorable results were demonstrated in patients (191).

**Figure 2:**
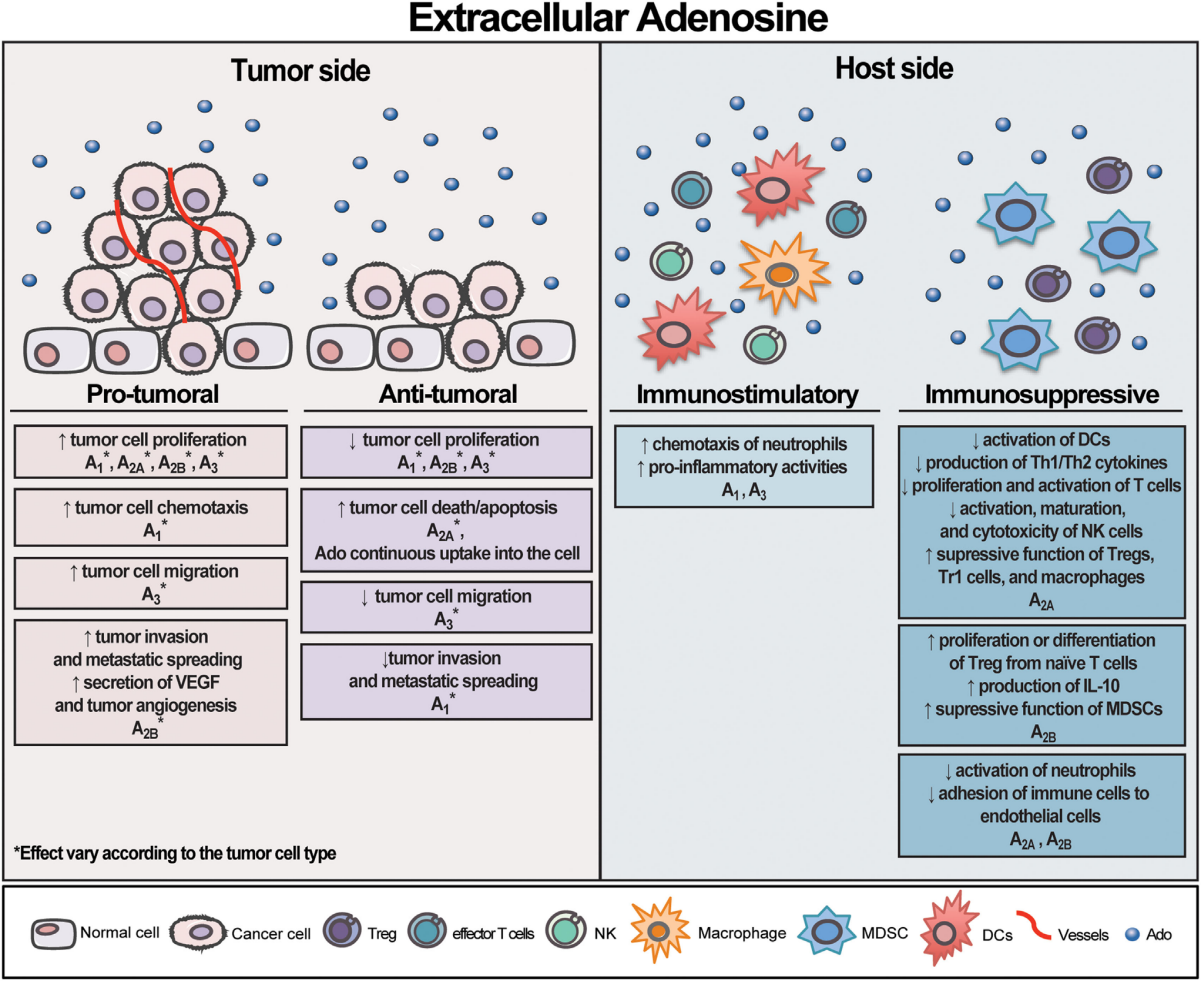
Schematic illustration exhibiting extracellular adenosine (eAdo) opposing effects on tumor and host side. Likewise extracellular adenosine triphosphate (eATP), eAdo can exert distinct and contrasting effects on both tumor and host cells depending on the cell type and receptor activated. eAdo can also promote tumor cell death *via* its continuous uptake into the cell. As depicted by eATP, the sum of eAdo levels, the group of P1 receptor subtypes, and CD39/CD73 expression by tumor and immune cells in the tumor microenvironment will dictate the final effect on tumor growth. Overall, eAdo is a potent immunosuppressive nucleoside, mostly inhibiting immune cell responses.

Rather than acting through P1 receptors, eAdo can also promote tumor cell death *via* its continuous uptake into the cell (52). Our group demonstrated that Ado formed from eATP degradation is the main factor responsible for apoptosis induction in human cervical cancer cells. Accordingly, eAdo transported into the cell through the nucleoside transporters leads to AMPK activation, p53 increase, PARP cleavage, and autophagy induction, culminating in cell death (52). Similar results were also reported in human gastric cancer cells (192), malignant pleural mesothelioma cell (193), mouse neuroblastoma cells (194), astrocytoma cells (195), and human epithelial cancer cells originating from breast, ileum, colon, and ovary (89, 196), bringing a distinct insight into the Ado effect on the tumor side. An overview of eAdo effect on tumor and host side is illustrated in Figure 2.

## Purinergic Signaling as Potential Target for Cancer Therapy

As depicted alongside this review, purinergic signaling has a major role in controlling tumor growth, survival, and progression, not only by acting on tumor cells but also by modulating the immune system and the interaction of tumor and immune cells in the TME (24). Therefore, many potential targets involving ATP and Ado signaling has emerged as attractive candidates for cancer therapy. In this topic, we will discuss recent findings in this field highlighting P2X7, CD39, CD73, and A_2A_ receptor targeting therapy to restrain tumor progression *in vivo* models and in patients.

### Targeting P2X7 Receptor in Cancer Therapy

As discussed earlier, the P2X7 receptor has contrasting effects when activated on the tumor or the host cells, potentiating or inhibiting tumor growth—depending on the level of stimulation—while boosting inflammation, respectively. Evidence supporting P2X7 growth-promoting activity has increased recently, and it appears to result from a large number of effects, i.e., inducing the release of immunosuppressive molecules by MDSCs and promoting VEGF release, angiogenesis, and tumor cell proliferation (23, 100). On the other hand, P2X7 receptor seems to restrain tumor growth by promoting DC/cancer cell interaction, cytokine release, chemotaxis, and infiltration of immune cells in the TME (53). Therefore, both strategies either stimulating or blocking P2X7 receptor have been studied to hinder cancer growth (46, 197).

P2X7 receptor overstimulation by using high levels of eATP was the first attempt to increase tumor cell death through its known apoptotic/necrotic function. Administration of very high levels of ATP (25 and 50 mM) effectively reduced the growth of hormone-refractory prostate cancer and melanoma tumors *in vivo*, respectively (198, 199). However, these studies were performed in nude athymic mice, therefore excluding a role for the immune system on this antitumor effect. eATP acting exclusively through P2X7 receptor also inhibited colon carcinoma and melanoma tumor growth in C57BL/6 wild-type mice, by perturbing the balance between two signaling axes—P2X7-PI3K/AKT and P2X7-AMPK-PRAS40-mTOR—and promoting tumor cell death through autophagy (48). Again, this result was focused on the stimulation of the tumor P2X7 receptor, and no mention to the host counterpart was reported. Regardless of these promising results, three clinical trials fail to demonstrate a beneficial impact by using exogenous ATP to treat cancer in patients, being an improvement of the quality of life the only positive effect demonstrated (200–202). Besides eATP, the use of P2X7 receptor agonists, such as BzATP and ATPγS, has also been employed to delay tumor growth, but once more, only the effect on the P2X7 receptor tumor side was evaluated (203, 204). Accordingly, BzATP inhibited the formation of DMBA/TPA-induced skin papillomas and carcinomas in wild-type FVB mice (203), while ATPγS decreased the tumor growth and metastasis of mouse mammary carcinoma cells in wild-type C57BL/6 mice (204).

P2X7 receptor activation through the eATP released from the irradiation and chemotherapy has also an important role in controlling tumor response to those treatments (205–207). In glioblastoma, P2X7 receptor expression by tumor cells dictated patient response to radiotherapy (208). Accordingly, high levels of P2X7 receptor are associated with good prognosis and increased glioma radiosensitivity. Moreover, P2X7 silencing prevents tumor response to radiation in an *in vivo* model of glioblastoma, reinforcing that functional P2X7 expression is crucial for an efficient radiotherapy response (208). Likewise, eATP acting *via* P2X7 receptor on DCs is determinant for the chemotherapy-induced ICD, stimulating host-specific immune responses (206, 207). We recently showed the importance of P2X7 receptor overactivation in colon cancer cells to potentiate chemotherapy cytotoxicity (209). According to our data, hyperthermia—by influencing plasma membrane fluidity—boosted P2X7 functional responses to eATP, leading to maximal tumor cell death, mainly in association with chemotherapy drugs. Therefore, P2X7 hyperactivation by hyperthermia might be used as an adjunct therapy in the treatment of cancer.

Tumor P2X7 receptor expression and activation and its impact on cancer proliferation have long been investigated. However, two recent studies also demonstrated a critical role for the host P2X7 receptor in stimulating the antitumoral immune response and restraining the tumor growth (47, 79). Correspondingly, animals with host genetic deletion of P2X7 were not able to mount an effective host inflammatory response, reporting reduced cell infiltration at the tumor bed, accelerated tumor growth, and metastatic spreading in comparison to the wild-type group.

Although the overstimulation of P2X7 receptor with agonists appears to be the most logical strategy to decrease tumor proliferation, by inducing both tumor cell death and antitumor immune response, recent studies have been demonstrated that blocking P2X7 receptor activation is more efficacious in preventing tumor growth, mainly in those cancers in which P2X7 receptor is overexpressed (28, 46, 47, 100). Administration of P2X7 inhibitors and antagonists has been shown to decrease cancer cell growth or spreading in animal models of colon (100), breast (115) and ovarian carcinoma (210), neuroblastoma (101), melanoma (47, 100), and glioma (211).

Several inhibitors and antagonists have been used to block P2X7 receptor in tumor cells, including oxidized-ATP (100, 212), BBG (210), AZ10606120 (47, 100, 101), A740003 (47, 101), A438079 (115), and also P2X7 blocking antibodies (115). A recent phase I clinical trial using anti-P2X7 antibody to treat basal cell carcinoma demonstrated exciting results and showed that 65% of patients respond to the treatment and had a significant reduction on the lesion area (213). The authors support the use of antibodies against P2X7 receptor as a safe and well tolerable treatment for BBC.

An important point to be considered is that the use of P2X7 receptor antagonists have been shown to demonstrate strong anticancer effects in immune-competent mice expressing P2X7 in both tumor and host side (47, 100), suggesting that blocking P2X7 on the tumor side is critical to the final antitumor action, despite the mild immunosuppressive effect due to inhibition of the P2X7 on the host side (53). Regardless, more studies investigating the P2X7 receptor function in host/tumor interactions, and their impact on tumor growth will indicate the feasibility of using P2X7 as a new target in cancer therapy.

### Blocking CD39 Activity—First Step to Inhibit Ado Formation and Restore Antitumor Immune Response

The conversion of eATP to Ado, either in physiological or pathological conditions, is mainly coordinated by the sequential activity of CD39 and CD73. In the TME, those enzymes will affect tumor growth according to their ability to produce Ado and therefore trigger an immunosuppressive signaling (24, 37).

Increased expression of CD39 has been widely reported in several tumors, such as medulloblastoma (214), sarcoma (215), HCC (216), pancreatic cancer (217), colorectal cancer (218, 219), gastric cancer (216), and endometrial cancer (220); as well as in infiltrating immune cells (216, 221–224) and tumor endothelial cells (216, 225), influencing tumor growth, metastasis and angiogenesis. As an example, expression of CD39 by Tregs plays a permissive role in a mouse model of hepatic metastasis by inhibiting NK cell antitumor immunity and contributing to tumor immune escape (226).

Therefore, strategies to block CD39 activity and Ado generation has become a new approach to avoid Ado immunosuppressive effects and restores the antitumor responses (36). So far, few approaches targeting CD39 by using pharmacological inhibitors, genetic deletion or antibodies have been rendered promising results (215, 224, 226, 227). As reported in the literature, blocking CD39 activity by using the inhibitor ARL67156 partially overcomes T cell hyporesponsiveness in a subset of patient samples with follicular lymphoma (224). In the same line, CD39 blockage with both inhibitor (ARL67156) and antibody (OREG-103/BY40) increased T cells and NK cell-mediate cytotoxicity against SK-MEL-5 melanoma cells (228). In an *in vivo* model, injection of POM1, a pharmacological CD39 inhibitor, was able to limit B16-F10 melanoma and MCA 38 colonic tumor growth at the same rate as demonstrated in animals CD39^−/−^ (226). Indeed, CD39 deletion inhibited metastatic melanoma and colonic growth in the liver as well as decreased tumor angiogenesis (226). Similarly, CD39 deletion abrogated B16-F10 melanoma and LLC lung carcinoma tumor growth, angiogenesis, and pulmonary metastases in mice (227). In another study, treatment with a specific anti-CD39 antibody significantly improved survival in a lethal metastatic patient-derived sarcoma model (215).

Altogether, these studies indicate that blocking Ado formation through targeting CD39 is a promising strategy in cancer therapy not only for boosting the antitumor immune response (immunotherapy) but also for blocking tumor angiogenesis (antiangiogenic therapy). However, future studies involving the use of anti-CD39 antibodies will provide supportive insights into the potential clinical application of CD39-targeting therapy in oncology (36).

### Inhibiting CD73 Activity—Second Step to Block Ado Formation and Improve Antitumor Immune Response

CD73 is a 5′ ectonucleotidase enzyme that degrades extracellular AMP—derived from the ATP metabolism—to Ado (37). As mentioned earlier, the sequential enzymatic activity of CD39 and CD73 is the main pathway for the generation of Ado in the tumor interstitium. In this context, CD73-derived Ado exerts many immunosuppressive effects to attenuate antitumor immunity (122). Likewise CD39, CD73 is expressed by cancer cells, regulatory immune cells, and the vasculature, therefore affecting tumor growth, metastasis and angiogenesis (36).

Elevated CD73 expression has been reported in several types of human cancers such as glioma (229–231), head and neck (128), melanoma (232), thyroid (233), breast (234–238), pancreas (239), colon (219, 240), bladder (241, 242), ovarian (243), prostate (244), and leukemia (126), being positively correlated with poor prognosis. In addition to tumor-derived CD73, host CD73 also negatively regulates tumor immunity (245). Accordingly, both hematopoietic and nonhematopoietic expression of CD73 is important to promote tumor immune escape. For example, Tregs-derived CD73 contributed to their immunosuppressive effects (245), while enzymatic activity of CD73 on tumor-associated endothelial cells restricted T cells homing to tumors (127). Altogether, these data suggest that both tumor and host CD73 cooperatively protect tumors from the immune system response, favoring cancer growth and spreading. Supporting this assumption, studies performed with CD73-deficient mice showed that animals lacking CD73 have an increased antitumor immunity and are resistant to carcinogenesis (245–247). Therefore, targeting CD73 appears to be a useful therapeutic tool to treat cancer.

Many approaches using small molecules inhibitors such as ACPC and antibodies against CD73 have shown important antitumor and antimetastatic effects in various preclinical models of melanoma (127, 245, 246, 248), fibrosarcoma (247), breast (125, 134, 235, 249, 250), prostate (247), and ovarian cancer (123). Those effects are mainly attributed to the immune-stimulating activity of CD73 blockage on host and tumor cells. However, a role for CD73 in controlling cancer cell proliferation independently of the immune system was also reported (251). Accordingly, CD73 gene-silencing in human tumor cells promoted cell-cycle arrest and apoptosis, decreasing cell growth rate in a xenograft tumor model.

Targeting CD73 has also been shown to suppress tumor angiogenesis (133, 134). Anti-CD73 therapy with monoclonal antibody significantly reduced tumor VEGF levels and abolished tumor angiogenesis in a mouse model of breast cancer (134). Accordingly, tumor-derived CD73 triggered VEGF production by tumor cells, while endothelial-derived CD73 promoted the formation and migration of capillary-like structures by endothelial cells, demonstrating that CD73 expression on tumor and host cells contribute to tumor angiogenesis.

A phase I clinical trial study is currently undergoing to test safety, tolerability, and antitumor activity of anti-CD73 mAb, MEDI9447, in cancer patients (NCT02503774) (Table 1). MEDI9447 is a selective, potent, and non-competitive inhibitor of CD73 that blocks both membrane-bound and soluble states of this enzyme (252). Preclinical data using mouse syngenic CT26 colon carcinoma tumor model showed that MEDI9447 inhibited tumor growth by promoting changes in both myeloid and lymphoid infiltrating leukocytes within the tumor interstitium (253). Among these changes, increasing number of CD8^+^ effector T cells and activated macrophages in the TME has been reported. In addition, mice treated with a combination of anti-CD73 and anti-programmed cell death protein (PD)-1 antibodies showed increased tumor rejection and survival rates when compared with mice treated with an individual antibody. Synergistic effects by combining CD73 blockade with other currently available anticancer agents, including anthracycline (254), radiation (160), anti-cytotoxic T-lymphocyte antigen (CTLA)-4 antibodies (255, 256), and anti-PD-1 antibodies (255) have also been reported and highlight the potential clinical application of CD73 target therapies in combination with other anticancer modalities to improve antitumor immune response as well as tumor death.

**Table 1:** Clinical trials currently underway that are testing the potential use of anti-CD73 mAb and A_2A_ antagonists alone or in combination with other immunotherapies to treat cancer.

Phase	Propose of study	Intervention	Condition	ID
I	Evaluate the safety, tolerability, pharmacokinetics, immunogenicity, and antitumor activity	Monotherapy: anti-CD73 mAb (MEDI9447) or Combination: anti-CD73 mAb (MEDI9447) and anti-PD-L1 mAb (MEDI4736)	Advanced solid tumors	NCT02503774
I/Ib	Determine the safety, tolerability, feasibility, and preliminary efficacy	Monotherapy: adenosine (Ado) A_2A_ receptor antagonist (PBF-509) or Combination: Ado A_2A_ receptor antagonist (PBF-509) and anti-PD-1 mAb (PDR001)	Non-small cell lung cancer	NCT02403193
I/Ib	Study the safety, tolerability, and antitumor activity	Monotherapy: Ado A_2A_ receptor antagonist (CPI-444) or Combination: Ado A_2A_ receptor antagonist (CPI-444) and anti–PD-L1 mAb (atezolizumab)	Non-small cell lung cancer Malignant melanoma Renal cell cancer Triple negative breast cancer Colorectal cancer Bladder cancer Prostate cancer	NCT02655822

### Blocking A_2A_ Receptor—Alternative Approach to Restrain Ado Immunosuppressive Effect and Boost the Antitumor Immunity

Targeting the Ado receptor A_2A_ is also an alternative approach to block the Ado immunosuppressive effect and boost the antitumor immunity (36). As depicted earlier, A_2A_ receptor plays an important role in triggering Ado immunosuppressive activities in many immune subsets. Therefore, blocking Ado A_2A_ receptor with antagonist appears to be an attracting strategy, besides CD39 and CD73 inhibition, to increase innate and adaptive immune response against the tumor (153). Many studies have been shown the potential use of A_2A_ antagonists alone or in combination with other therapies to enhance antitumor immunity in preclinical models (125, 150, 257, 258). Combination therapies targeting both A_2A_ receptor and co-inhibitory molecules, such as CTLA4 and PD-1, have shown synergistic effects (256, 257, 259). Coadministration of A_2A_ antagonist with anti-CTLA4 mAb marked inhibited tumor growth and enhanced antitumor immune responses in a mouse melanoma model (256). Moreover, dual blockade of A_2A_ receptor and PD-1 significantly reduced CD73^+^ tumor growth and metastasis spreading as well as prolonged mice survival (257, 259). The mechanism of the combination therapy was mainly dependent on NK cells, CD8^+^ T, cells and IFN-γ. Importantly, the overexpression of CD73 by tumor cells was critical for the efficacy of the combined therapy, suggesting that CD73 might be a potential biomarker for the selection of patients undergoing this method of treatment. Supporting this statement, co-inhibition of CD73 and A_2A_ receptor by either gene deletion or pharmacological therapy limited tumor initiation, growth, and metastasis *in vivo* (260). In the double knockout (KO) mice, tumor control required CD8^+^ T-cell and IFN-γ production within the core of tumors, while therapeutic activity of CD73 antibodies depend on Fc receptors binding. Interestingly, A_2A_ single KO mice showed a significant upregulation of CD73 expression in tumor cells and endothelial cells, suggesting that CD73 overexpression might be a mechanism of escape and resistance to monotherapy with A_2A_ antagonists. So far, two clinical trials (phase I) are currently underway to evaluate safety, tolerability, and antitumor activity of A_2A_ antagonists as a single agent and in combination with PD-1/PD-L1 inhibitors in patients (NCT02403193 and NCT02655822) (Table 1). Therefore, associating A_2A_ antagonist with other checkpoint blockade inhibitors appears to be a promising strategy to improve patient survival and yet many researchers have pointing the anti-adenosinergic signaling as the next-generation target in immuno-oncology.

## Conclusion

**Figure 3:**
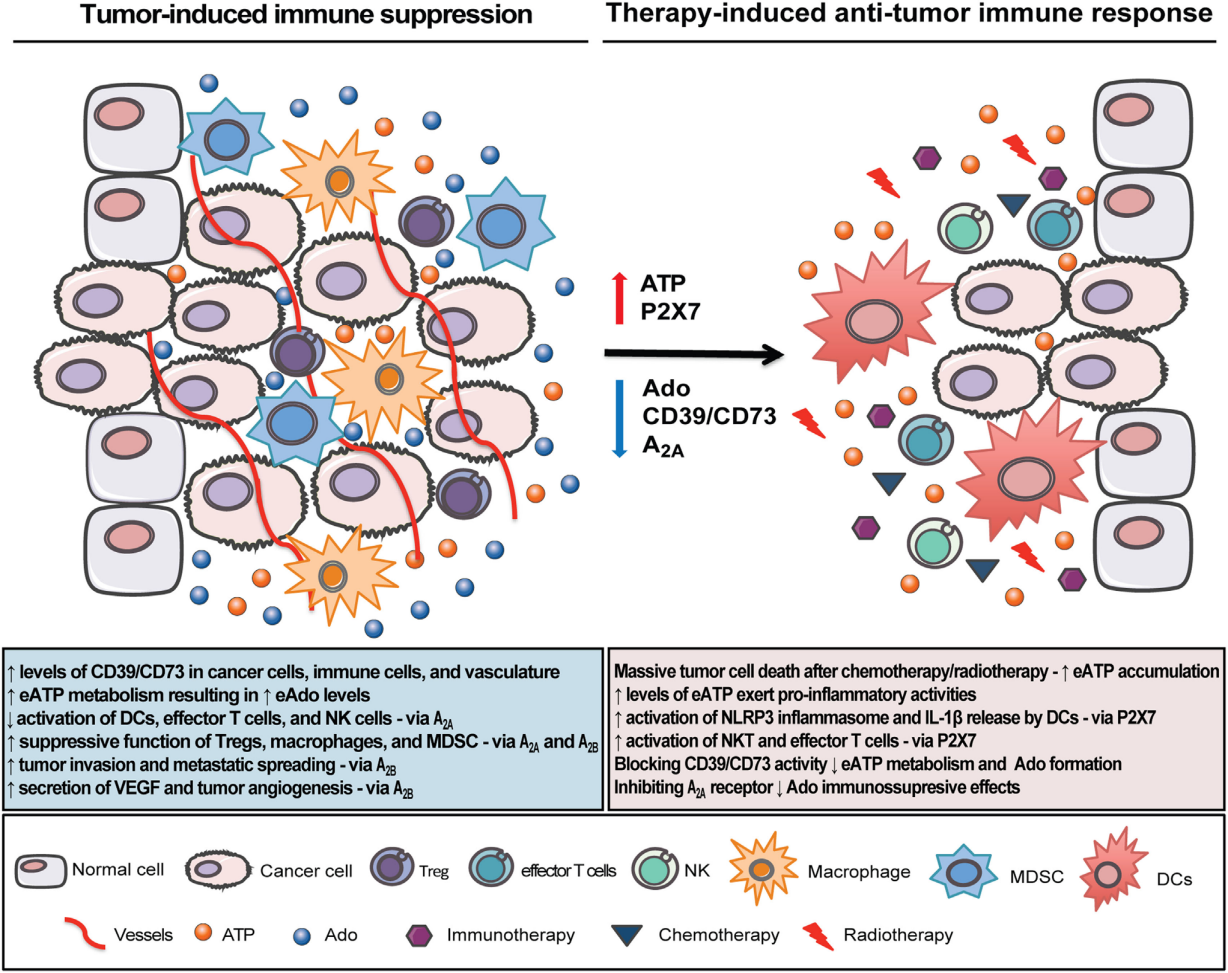
Therapeutic strategies to overcome tumor immune escape and boost cancer immunosurveillance in the tumor microenvironment (TME). In the inflammatory TME, tumor and immune cells interact to produce a favorable immunosuppressive microenvironment. Extracellular adenosine triphosphate (eATP), a pro-inflammatory mediator, accumulates in the TME, but it is rapidly converted to the immunosuppressive factor adenosine (Ado) *via* the sequential enzymatic activity of CD39 and CD73. Ado acting through A_2A_ and A_2B_ receptors inhibits dendritic cells (DCs), NK, and effector T cells activation while it enhances the suppressive function of Tregs, macrophages, and myeloid-derived suppressor cell (MDSC). Strategies by targeting Ado formation, i.e., by blocking CD39/CD73 enzymes and Ado receptors (mainly A_2A_) will build up eATP concentration and improve the antitumor immune response. Specifically on DCs, eATP acting through P2X7 receptor will trigger NLRP3 inflammasome activation and IL-1β release with consequent stimulation of CD8^+^ and CD4^+^ lymphocyte-mediated antitumor response, which is a critical step for the efficacy of chemotherapy and radiotherapy. Therefore, combining purinergic-targeting therapies with other anticancer modalities may be a new strategy to overcome immune escape, potentiate antitumor immune response, and consequently restrain tumor growth.

Despite its complexity and contradictory effects, purinergic signaling has emerged as a novel targetable therapy to improve other anticancer modalities and cannot be underestimated considering its role in carcinogenesis. Strategies by blocking Ado formation and its immunosuppressive effects in the TME favoring eATP accumulation, and its pro-inflammatory effects appears to be the most promising approach to maximize the efficacy of other therapies such as immunotherapy, radiotherapy, and chemotherapy (Figure 3). However, considering the multifaceted effects of eATP and Ado in the TME, where host immune and stromal cells as well as tumor cells are modulated in different ways, choosing the most feasible purinergic target will be a challenging task. Ongoing and upcoming clinical trials will hopefully identify the best combinatorial approach to boost antitumor immune response and successfully restrain tumor growth.

## Author Contributions

PM, RC-S, and LS wrote the article. All the authors contributed to the study conception and design, and critically revised the manuscript.

## Conflict of Interest Statement

The authors declare that the research was conducted in the absence of any commercial or financial relationships that could be construed as a potential conflict of interest.
